# Salicylic Acid Stimulates Defense Systems in *Allium hirtifolium* Grown under Water Deficit Stress

**DOI:** 10.3390/molecules27103083

**Published:** 2022-05-11

**Authors:** Peyman Yousefvand, Yousef Sohrabi, Gholamreza Heidari, Weria Weisany, Andrea Mastinu

**Affiliations:** 1 Department of Agronomy and Plant Breeding, Faculty of Agriculture, University of Kurdistan, Sanandaj 66177, Iran; yousefvandpeyman@gmail.com (P.Y.); g.heidari@uok.ac.ir (G.H.); 2 Department of Agriculture and Food Science, Science and Research Branch, Islamic Azad University, Tehran 14778, Iran; weria.wisany@gmail.com; 3 Department of Molecular and Translational Medicine, University of Brescia, 25123 Brescia, Italy

**Keywords:** membrane stability, growth regulator, water deficit, chlorophyll content, *Allium hirtifolium*, secondary metabolites

## Abstract

Nowadays, the use of the growth regulator salicylic acid for improving a plant’s resistance to environmental stresses such as drought is increasing. The present study investigated the effect of salicylic acid on the physiological traits, antioxidant enzymes, yield, and quality of *Allium hirtifolium* (shallots) under drought conditions for three years (2016–2017, 2017–2018, and 2018–2019). The experiment was conducted as a split-plot based on a randomized complete block design with four repeats. Irrigation as the main factor in four levels of 100% (full irrigation), 75% and 50% of the plant water requirements with non-irrigation (dryland), and salicylic acid as the sub-factor in four levels of 0, 0.75, and 1 mmol, were the studied factors in this research. The combined analysis of three-year data showed that drought reduced leaf relative water content (RWC), membrane stability index (MSI), chlorophyll content, onion yield, and increased activity of antioxidant enzymes, proline content, tang, and allicin of shallots. Shallot spraying with salicylic acid improved leaf RWC, MSI, chlorophyll content, and onion yield. The highest yield of onion (1427 gr m^−2^) belonged to full irrigation and foliar application of 1 mmol salicylic acid. The lowest yield (419.8 gr m^−2^) belonged to plats with non-irrigation and non-application of salicylic acid. By improving the effective physiological traits in resistance to water deficit, salicylic acid adjusted the effects of water deficit on the yield of shallots. Foliar application of 1 mmol salicylic acid in dryland and irrigation of 50% of the plant water requirement increased onion yield by 15.12% and 29.39%, respectively, compared to the control treatment without salicylic acid.

## 1. Introduction

Medicinal plants are one of the most valuable plants that are nowadays used widely to cure different diseases and improve human health [1,2]. *Allium hirtifolium* is a perennial plant from the Liliaceae family that has underground tuber and is from by-products of pastures that grow in high and natural habitats [3]. *A. hirtifolium* has about 0.06–1% essential oil in chemical composition, including allicin-alicathin 1, 2, allyl propyl-disulfide, and two sulfur compounds. *A. hirtifolium* is used in the food and pharmaceutical industries and is of considerable economic importance [4]. Due to the excessive use of this plant in natural habitats, research on the process of domestication and high production of this plant in agricultural farms in order to preserve natural resources and prevent their destruction is necessary and very important [5]. As the natural production of Persian *A. hirtifolium* does not meet the needs of food and pharmaceutical industries, it may become extinct in the future. Thus, researchers believe that the cultivation and production of endangered herbs in agricultural ecosystems are necessary [6]. However, the development of agriculture and efforts to increase the production and yield of crops needed by humans have always faced many limitations, one of the most influential environmental limiting factors on crop yield, abiotic stresses [7]. One of these limitations is the reduced rainfall and water shortage available in many parts, including Iran. Drought is the most common and important environmental stress limiting the yield of many crops and medicinals, especially in arid and semi-arid regions [8,9], which reduces plants yield by more than 50% on average. Drought stress causes a broad range of physiological changes and impairments of metabolic processes, which result in the accumulation of reactive oxygen species (ROS) [10,11].

Drought stress reduces plant water potential, produces reactive oxygen species (ROS), damages cell membranes, inhibits electron transfer, destroys chloroplast and thylakoid membrane structures, and degrades and reduces photosynthetic pigments [9,11,12]. It also reduces the photosystem function and finally decreases photosynthesis [13]. Plant cells produce free radicals under stress conditions, which causes toxicity in the plant. Antioxidants cause the alleviation of the ROS toxicity obtained from induced stress in plants and protect plant cells from damage [14].

Salicylic acid (SA) and its derivatives, acetyl salicylic acid (ASA) and methyl salicylate (MeSa), are plant hormones that play important roles in a wide range of physiological processes, from seed germination to flowering and fruit ripening. However, the most studied roles have been their effects on inducing plant defense systems against different biotic and abiotic stresses [15]. In particular, SA regulates physiological processes in plants, reduces stress’ side effects and can improve the adverse effects of stress [11]. SA successfully protects membranes and cell organs, including the cell protein synthesis machine and the structure of proteins and enzymes, by reducing oxidative stress, increasing the amount of proline, and reducing their oxidation or degradation. Previous research has shown that spraying SA decreases lipid peroxidation and H_2_O_2_ levels while also protecting cell membranes, devices, and photosynthetic pigments by improving antioxidant capacity, including carotenoids. SA regulates plants’ physiological and biochemical properties under abiotic stresses and improves their resistance to diseases [16].

According to the high medicinal value of *A. hirtifolium* among medicinal plants and the need to cultivate this product to preserve natural resources and reduce environmental damage, research on this plant is fundamental. Therefore, finding appropriate solutions to reduce the effects of drought stress and providing the possibility of increasing crop yield in these conditions can be essential. Despite all the mentioned above cases, not much effort has been made to the cultivation and investigation of the possibility of producing this medicinal plant in water-deficit regions. Additionally, according to our information, no report has been presented to evaluate the effect of SA on the growth and yield of *A. hirtifolium* under drought stress conditions. Therefore, the purpose of the present study was to investigate the possibility of producing an acceptable product under water-deficit conditions. Furthermore, the present study is the first effort in determining the effect of different levels of SA on reducing and modulating the effects of water deficit on physiological traits and yield of *A. hirtifolium* medicinal plant.

## 2. Results

### 2.1. Relative Leaf Water Content (RWC)

An analysis of variance of experimental data showed that the triple interaction of year, irrigation levels, and SA consumption and the dual interaction of irrigation and SA levels on leaf RWC were significant at a 1% probability level (Table 1).

The results of comparing the mean indicate that the application of drought stress and increasing its intensity caused a significant decrease in the relative water content of the leaf (Table 2), so that every three years of the experiment and at all levels of application or non-application of salicylic acid, the lowest amount of this trait was 50% of the plant water requirement in dryland and irrigation conditions, respectively.

Irrigation treatments 75% of the plant water requirement and especially full irrigation had the highest RWC of the leaf, and their differences with the other two irrigation treatments were quite significant (Table 3). In all three years, foliar application of the plant with SA in dryland conditions and irrigation level of 50% of the plant water requirement improved the RWC of the *A. hirtifolium* plant. Application of this substance in conditions of full irrigation and irrigation at 75% of plant water requirement did not have a significant effect on the RWC of the leaf, so the highest RWC of the leaf with 64.23% was obtained in the first year and full irrigation and non-use of SA. The lowest value of this trait, with an average of 40.05%, was allocated to dryland conditions and lack of foliar application in the first year (Table 3). In all three years, the application of 1 mM SA in *A. hirtifolium* under dryland conditions increased the RWC by 20.34%, 20.14%, and 9.97%, respectively (Table 3).

### 2.2. Membrane Stability Index (MSI)

The analysis of variance results displayed that the triple interaction of SA, irrigation, and year, as well as the dual interaction of SA in irrigation and irrigation levels in the year on the MSI, were significant at the level of one percent probability (Table 1). Comparing the mean of treatments was found that in all three years of the study and at all levels of SA application, the application of drought stress and increasing its intensity significantly reduced the percentage of membrane stability. In all three years, the highest percentage of membrane stability belonged to full irrigation. The plants under an irrigation level of 75% of the plant water requirement were next. The lowest membrane stability was obtained in plants under dryland conditions, which showed a significant difference with other irrigation levels. The use of SA for almost all concentrations in water deficit conditions increased the amount of this trait, and this increase was more pronounced in the second year in plants under dryland conditions. However, no significant difference was observed between the non-use of SA and its use in most cases (Table 3). Under full irrigation conditions, in every three years of experimentation, applying only 1 mM concentration of this substance increased the membrane stability in *A. hirtifolium*, and other concentrations of SA decreased the membrane stability, although these changes were not significant. Among the studied treatments, the highest amount of MSI with an average of 97.14% was related to full irrigation (100% water requirement of the plant) and spraying of 1 mM SA in the second year. The lowest value of this trait, with an average of 36.51%, was observed in the treatment of non-irrigation and non-consumption of SA in the third year, which, with other levels of application of SA in this level of irrigation and the same year and the first year, had the lowest membrane stability (Table 3).

### 2.3. Proline

**Figure 1:**
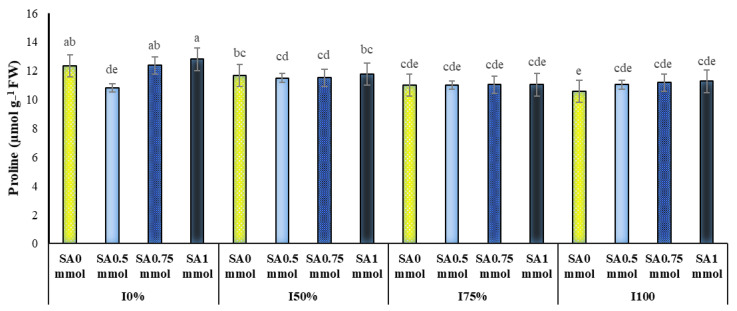
Dual interaction of different levels of irrigation (I0 = no irrigation, I50% = 50%, I75% = 75% and I100% = 100% water requirement of the plant) and salicylic acid application concentrations (SA0 = non-application, SA0.5 = 0.5 mmol, SA0.75 = 0.75 mmol and SA1 = 1 mmol) on the proline content of *A. hirtifolium*. Columns designated by the same letter are not significantly different at the *p* ≤ 0.05 level as determined by the LSD test.

The results of the analysis of the table of variance revealed that the simple effect of year and the interaction of irrigation and SA levels on *A. hirtifolium* proline content were significant at the level of one percent probability (Table 1). Comparing the mean of the data in Table 2 indicates that the amount of proline in the first year was higher than the other two years. In the second and third years, this trait showed a relatively similar situation and was in a statistical group. A comparison of means showed that drought stress and foliar application of SA increased the amount of proline in the plant. The highest amount of proline (12.80 μmol g^−1^ fresh leaf weight) was in the treatment of non-irrigation and foliar application of 1 mmol SA. The lowest amount of proline (10.58 μmol g^−1^ fresh leaf weight) was obtained in the complete irrigation treatment (100% water requirement of the plant) and non-use of SA (Figure 1).

### 2.4. Antioxidant Enzymes

#### 2.4.1. Superoxide Dismutase (SOD)

**Figure 2:**
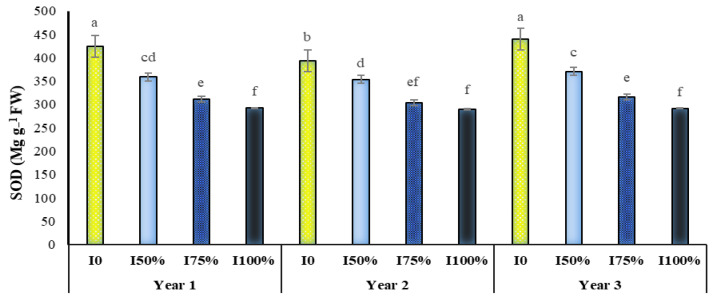
Dual interaction of experimental years (2016–2017, 2017–2018 and 2018–2019) and different levels of irrigation (I0 = no irrigation, I50% = 50%, I75% = 75% and I100% = 100% water requirement of the plant) on the activity of superoxide dismutase (SOD) enzyme in *A. hirtifolium*. Columns designated by the same letter are not significantly different at the *p* ≤ 0.05 level as determined by the LSD test.

**Figure 3:**
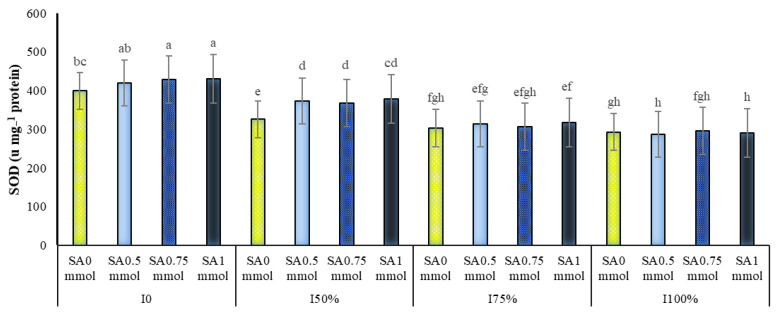
Dual interaction of different levels of irrigation (I0 = no irrigation, I50% = 50%, I75% = 75% and I100% = 100% water requirement of the plant) and salicylic acid application concentrations (SA0 = non-application, SA0.5 = 0.5 mmol, SA0.75 = 0.75 mmol and SA1 = 1 mmol) on the activity of superoxide dismutase (SOD) enzyme in *A. hirtifolium*. Columns designated by the same letter are not significantly different at the *p* ≤ 0.05 level as determined by the LSD test.

The interactions of irrigation levels in year and irrigation levels in SA were significant on SOD activity at the probability level of 1% (Table 1). The highest activity of this enzyme was observed in all three years of research in plants under dryland conditions. Plants under irrigation conditions of 50% and 75% of plant water requirement were in the following ranks, and plants under full irrigation had the lowest enzyme activity. We classified all irrigation levels into different statistical groups based on SOD activity (in all three years) (Figure 2). A comparison of means showed that the application of drought stress and foliar plant application with SA increases the amount of the SOD enzyme (Figure 3). As shown in Figure 3, the occurrence of drought stress and its increase in intensity significantly increased the SOD enzyme activity. At all irrigation levels, the use of SA increased the activity of this enzyme in the plant compared to not using it. However, this increase was more pronounced in severe drought stress conditions (dryland and 50% of the plant’s water requirement) and was significant. Among the studied treatments, the highest amount of activity of this enzyme with 429.9 unit mg^−1^ protein belonged to non-irrigation and consumption of 1 mM SA and the lowest with 287 unit mg^−1^ protein to full irrigation (100% water requirement) and consumption 0.5 mM SA was allocated (Figure 3).

#### 2.4.2. Glutathione Peroxidase (GPX)

The results showed that the main effects of year and irrigation levels on GPX activity were significant at the level of 1% probability (Table 1). A comparison of means showed that the activity of GPX enzyme in *A. hirtifolium* grown in the first year was significantly higher than the activity of this enzyme in the other two years. The activity of this enzyme in plants grown in the second and third years was in a statistical group (Table 2). The results also showed that drought stress increased the activity of this enzyme (Table 2). The highest activity of this enzyme with 89.12 unit mg^−1^ of protein was related to non-irrigation treatment, and the lowest amount of this trait with 74.36 unit mg^−1^ of protein was obtained from full irrigation treatment (100% of plant water requirement) (Table 2). Plants under dryland conditions and levels of 50% and 75% water requirement of the plant had compared to full irrigation conditions had about 20, 14, and 8% higher GPX activity, respectively.

#### 2.4.3. Peroxidase (POD)

The analysis of variance showed that the triple interaction of irrigation, SA, and year levels on POD activity was significant at the level of one percent probability (Table 1). A comparison of the mean of the data showed that the occurrence of drought stress and foliar application of *A. hirtifolium* with SA increased the activity of this enzyme (Table 3). The occurrence of drought stress and its intensity increasing significantly increased the POD enzyme activity, so that the activity of POD increased in dryland conditions compared to full irrigation by 30% (Table 2). This enzyme’s highest activity was observed in all three years of research in plants under dryland conditions. Plants under irrigation conditions of 50% and 75% of plant water requirement were in the following ranks, and plants under full irrigation had the lowest enzyme activity. We classified all irrigation levels into different statistical groups based on POD activity in all three years (Table 3). In three years of research and at all levels of irrigation, SA application increased the activity of this enzyme compared to not using it. However, in most cases, this increase was not significant. Among the studied treatments, the highest activity of POD enzyme with 343.1 μmol min^−1^ mg^−1^ protein in the treatment of non-irrigation and spraying of 0.5 mM SA, and the lowest value of this trait with 213.3 μmol min^−1^ mg^−1^ protein full irrigation, and an application of 0.5 mM SA was obtained (Table 3).

### 2.5. Chlorophylls a, b and Total Content

**Figure 4:**
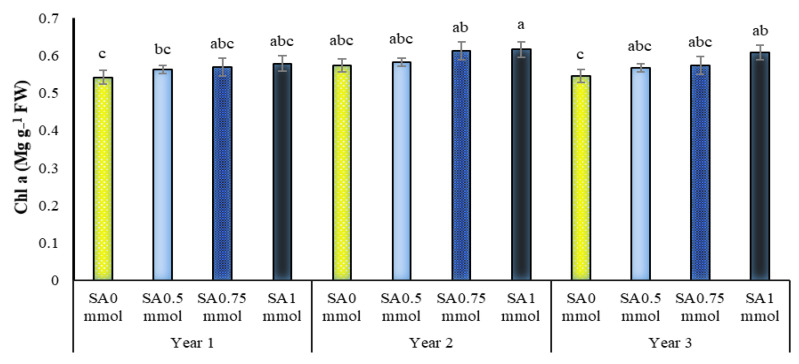
Dual interaction of experimental years (2016–2017, 2017–2018, and 2018–2019) and salicylic acid application concentrations (SA0 = non-application, SA0.5 = 0.5 mmol, SA0.75 = 0.75 mmol, and SA1 = 1 mmol) on chlorophyll a in *A. hirtifolium*. Columns designated by the same letter are not significantly different at the *p* ≤ 0.05 level, as determined by the LSD test.

The simple effect of irrigation levels and dual interactions of SA in the year on chlorophyll content a and triple interactions and all dual interactions on chlorophyll b content of *A. hirtifolium* were significant (Table 1). Comparing the means of the data in Table 2 shows that applying drought stress and increasing the severity of water shortage significantly reduced the chlorophyll ‘a’ content. The highest amount of this pigment (0.668 mg g^−1^ leaf fresh weight) belonged to plants under full irrigation, which was significantly superior to other levels of irrigation. The lowest amount of this trait (0.155 mg g^−1^ leaf fresh weight) was assigned to plants under dryland conditions, significantly different from other irrigation levels. There was no significant difference between irrigation levels of 75 and 50% of plant water requirement, and these two treatments were in a statistical group in terms of chlorophyll ‘a’ content. It was found that in all three years of research, the use of SA increased the chlorophyll ‘a’ content in *A. hirtifolium* (Figure 4). Among the studied treatments, the highest chlorophyll ‘a’ content (0.616 mg g^−1^ leaf fresh weight) was related to using 1 mM SA in the second year. The lowest value of this trait (0.542 mg g^−1^ leaf fresh weight) was observed in the absence of SA in the first year (Figure 4).

Comparison of means in Table 3 showed that in all three years of the study, the highest chlorophyll ‘b’ content at all levels of SA application belonged to the irrigation level of 100% of the plant water requirement (full irrigation) followed by 75% of the plant irrigation requirement. The application of drought stress and increasing its intensity caused a significant decrease in chlorophyll ‘b’ content, so that in three years, and at all levels of application or non-application of SA, the lowest amount of this trait was allocated to plants under dryland conditions, which, in most cases, were in a statistical group with plants treated by irrigation of 50% of the plant water requirement (Table 3). In all three years, the foliar application of SA at all irrigation levels, especially in dryland conditions and at an irrigation level of 50% of the plant water requirement, improved the chlorophyll ‘b’ content in *A. hirtifolium*. However, there was no significant difference between the application and non-application of SA in some cases (Table 3). The highest amount of chlorophyll ‘b’ in all levels of SA application was related to full irrigation treatment in the first and second years. The lowest amount of this pigment in all three years was related to the control treatment (non-use of SA) under drought stress conditions (dryland and 50% water requirement) (Table 3).

**Figure 5:**
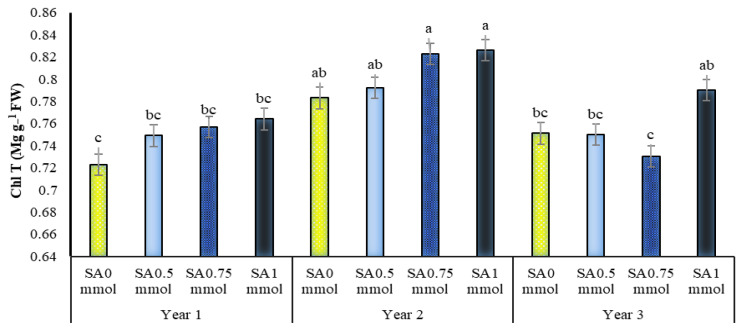
Dual interaction of experimental years (2016–2017, 2017–2018, and 2018–2019) and salicylic acid application concentrations (SA0 = non-application, SA0.5 = 0.5 mmol, SA0.75 = 0.75 mmol and SA1 = 1 mmol) on total chlorophyll content in *A. hirtifolium*. Columns designated by the same letter are not significantly different at the *p* ≤ 0.05 level as determined by the LSD test.

The present study results showed that the main effects of year, irrigation levels and SA, and dual interaction SA in the year on total chlorophyll content were significant (Table 1). Drought stress reduced total chlorophyll content in *A. hirtifolium* so that, with increasing stress intensity, total chlorophyll decreased more. There was a significant difference between all irrigation levels (Table 2). Foliar spraying with 1 mmol SA in all three years of the experiment, especially in the second year, improved the total chlorophyll content in the *A. hirtifolium*. However, there was no significant difference between the use and non-use of SA (Figure 5). The highest total chlorophyll content (0.826 mg g^−1^ fresh leaf weight) was related to using 1 mmol SA in the second year. In the first year, the lowest value for this trait (0.723 mg g^−1^ fresh weight of leaves) was obtained in the absence of SA, which resulted in a statistical association with all treatments of SA in the same year and non-application and applications of 0.5 and 0.75 mmol in the third year (Figure 5).

### 2.6. Carotenoid Content

The results of an analysis of variance showed that the triple interaction of SA, irrigation, and year, as well as the dual interactions of SA in irrigation, SA in the year, and irrigation levels in the year on carotenoid content, were significant at the level of 1% probability (Table 1). It was found that in all three years of the study, the application of drought stress and increasing its intensity caused a significant decrease in carotenoid content. In all three years, the highest carotenoids content belonged to full irrigation. The lowest quantity of carotenoids was obtained in plants under non-irrigation conditions, significantly differing from other irrigation levels. The use of almost all concentrations of SA in conditions of water deficiency increased the values of this trait (Table 3). However, in dryland conditions, in all three years, the application of concentrations of 0.75 and 1 mM of this material increased the content of carotenoids in *A. hirtifolium* more than the control treatment without SA. Among the studied treatments, the highest quantity of carotenoids (0.377 mg g^−1^ fresh leaf weight) was related to full irrigation (100% water requirement of the plant) and 1 mM spraying of SA in the second year. In the second year, when no irrigation was used and no SA was applied, this trait was the lowest (0.222 mg g^−1^ leaf fresh weight). Other levels of SA application in this level of irrigation in the third year and 0 and 0.5 mm levels of SA in the first year also had the lowest quantity of carotenoids (Table 3).

### 2.7. Yield

**Figure 6:**
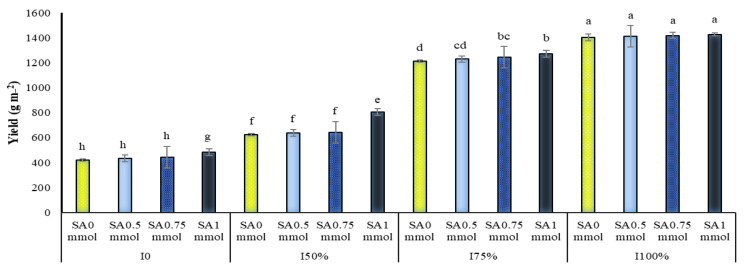
Dual interaction of different levels of irrigation (I0 = no irrigation, I50% = 50%, I75% = 75%, and I100% = 100% water requirement of the plant) and salicylic acid application concentrations (SA0 = non-application, SA0.5 = 0.5 mmol, SA0.75 = 0.75 mmol and SA1 = 1 mmol) on *A. hirtifolium* onion yield. Columns designated by the same letter are not significantly different at the *p* ≤ 0.05 level as determined by the LSD test.

The analysis of variance showed that the dual interaction of irrigation levels in SA and the dual interactions of SA application in the year and irrigation levels in the year on *A. hirtifolium* onion yield were significant (Table 1). A comparison of means showed that drought stress and increasing its intensity significantly reduced the yield of *A. hirtifolium* onions. The highest onion yield at all levels of application and non-application of SA belonged to plants under full irrigation treatments, 75% and 50% of the plant water requirement, respectively. The lowest yield was observed in dryland conditions. In the present study, different irrigation levels were classified into different statistical groups regarding onion yield (Figure 6).

**Figure 7:**
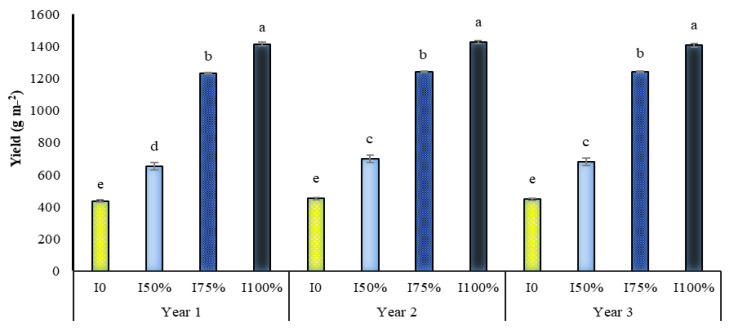
Dual interaction of experimental years (2016–2017, 2017–2018, and 2018–2019) and different levels of irrigation (I0 = no irrigation, I50% = 50%, I75% = 75% and I100% = 100% water requirement of the plant) on the onion yield. Columns designated by the same letter are not significantly different at the *p* ≤ 0.05 level as determined by the LSD test.

**Figure 8:**
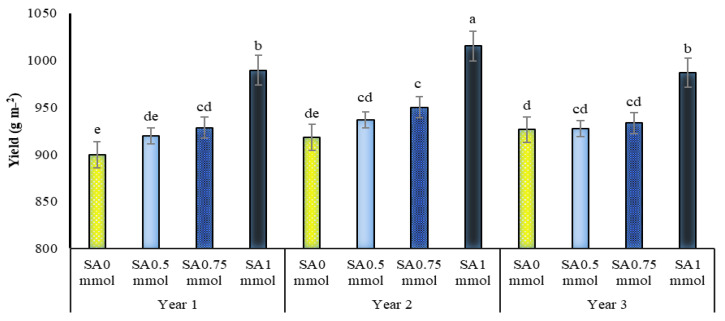
Dual interaction of experimental years (2016–2017, 2017–2018, and 2018–2019) and salicylic acid application concentrations (SA0 = non-application, SA0.5 = 0.5 mmol, SA0.75 = 0.75 mmol and SA1 = 1 mmol) on *A. hirtifolium* yield. Columns designated by the same letter are not significantly different at the *p* ≤ 0.05 level as determined by the LSD test.

At all irrigation levels, the use of SA, especially at a concentration of one mmol, increased the yield of *A. hirtifolium*, and this concentration of SA, especially under stress, significantly increased the yield of onions. As shown in Figure 6, applying this concentration of SA in dryland conditions and 50% of the plant water requirement compared to not using it increased onion yield by 15.12 and 29.39%, respectively (Figure 6). The highest yield of onion with 1427 g m^−2^ belonged to full irrigation and foliar application of 1 mmol SA. The lowest yield with 419.8 g m^−2^ was related to no irrigation and no foliar application SA (Figure 6). Comparing the mean of the dual interaction between the experimental years and irrigation levels showed that drought stress reduced the yield of onion in *A. hirtifolium* in all three years of the study. With increasing stress intensity, the decrease in the amount of this trait was more significant. In all three years of research, the highest yield of onions was obtained in plants under full irrigation, and plants irrigated with 75% and 50% treatments were in the following ranks, respectively. The lowest yield in dryland conditions was obtained (Figure 7). It was found that in all three years of the study, the use of SA increased onion yield. In all three years, increasing the concentration of application of this substance increased the yield of *A. hirtifolium* onion. However, the highest amount of onion yield belonged to the use of 1 mmol SA. The lowest amount of this trait was obtained in plants under the conditions of non-use from SA (Figure 8).

### 2.8. Allicin

The triple interaction of SA application, irrigation, and year, as well as the dual interaction of irrigation and SA levels on allicin content at the level of 1% probability, were significant (Table 1). A comparison of means showed that drought stress increased the amount of allicin in *A. hirtifolium*. The higher the stress intensity, the more allicin was produced in the plant, so that in all three years of research, the highest amount of allicin was produced in dryland plants. Plants under irrigation of 50% and 75% of plant water requirement were in the following ranks, respectively, and the lowest amount of allicin was obtained in plants under full irrigation conditions. There was no significant difference between plants under full irrigation and 75% of plant water requirement in allicin produced. Plants under these two irrigation treatments were placed in a statistical group in all three years (Table 3). The use of SA in all three years reduced the amount of allicin production in the plant, which was observed at all irrigation levels. However, this reduction was not significant in most cases, and only in a few cases in the application of 1 mmol SA under dryland conditions and with an irrigation level of 50% of plant water requirement was this reduction significant (Table 3). The highest amount of allicin with 4.05 mg g^−1^ of fresh leaf tissue was obtained in treated plants with non-irrigation and non-use of SA. The lowest amount of this trait with 1.61 mg g^−1^ of fresh leaf tissue belonged to full irrigation treatment (100% water requirement of the plant) and use of 0.50 mmol SA (Table 3).

### 2.9. Pyruvate

**Figure 9:**
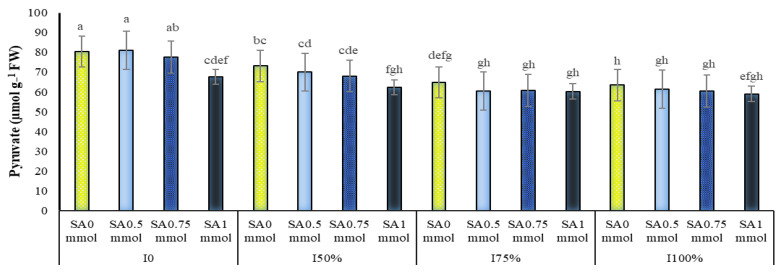
Dual interaction of different levels of irrigation (I0 = no irrigation, I50% = 50%, I75% = 75% and I100% = 100% water requirement of the plant) and salicylic acid application concentrations (SA0 = non-application, SA0.5 = 0.5 mmol, SA0.75 = 0.75 mmol and SA1 = 1 mmol) on the amount of pyruvate (sharpness) of *A. hirtifolium*. Columns designated by the same letter are not significantly different at the *p* ≤ 0.05 level as determined by the LSD test.

**Figure 10:**
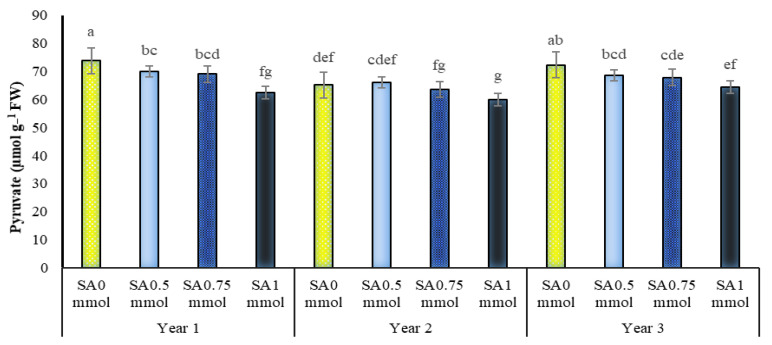
Dual interaction of experimental years (2016–2017, 2017–2018, and 2018–2019) and salicylic acid application concentrations (SA0 = non-application, SA0.5 = 0.5 mmol, SA0.75 = 0.75 mmol and SA1 = 1 mmol) on the pyruvate (sharpness) of *A. hirtifolium*. Columns designated by the same letter are not significantly different at the *p* ≤ 0.05 level as determined by the LSD test.

The dual interactions of irrigation with SA and SA with the year on the pyruvate of *A. hirtifolium* at the level of one percent probability were significant (Table 1). A comparison of means showed that drought stress and increasing its intensity increased the amount of pyruvate, and the use of SA decreased the amount of this trait (Figure 9). Providing sufficient moisture for *A. hirtifolium* reduced the amount of pyruvate of this plant, but the lack of water significantly increased the intensity. The highest pyruvate obtained in this study belonged to plants under dryland conditions, which showed significant differences with plants under other levels of irrigation at all levels of SA application. The application of SA, especially its concentration of 1 mmol in water shortage conditions (dryland and 50% of water requirement of the plant), caused a significant reduction in the pyruvate of *A. hirtifolium*. In full irrigation conditions and irrigation with 75% of plant water requirement, no significant difference was observed between different treatments of application or non-application of SA. Among all the treatments studied, the highest pyruvate with 81.06 μmol g^−1^ of fresh leaf weight was related to non-irrigation and 0.5 mmol SA. The lowest value of this trait with 58.96 μmol g^−1^ of fresh leaf weight was related to complete irrigation treatment (100% plant water requirement) and the use of 1 mmol SA (Figure 9). The results showed that the foliar application of 1 mmol SA in irrigation treatments with 50% water requirement of plant and non-irrigation reduced the *A. hirtifolium* pyruvate by 17.37 and 18.87 percentage, respectively (Figure 9). As shown in Figure 10, SA reduced the pyruvate of *A. hirtifolium*. Increasing the concentration of foliar application, the decrease in the amount of this trait was more noticeable so that the lowest amount of *A. hirtifolium* in each of the three years of research was observed in the consumption of 1 mmol SA, and the highest amount of this trait belonged to plants without SA (Figure 10).

## 3. Discussion

Drought stress and increasing its intensity caused a significant decrease in the relative water content of the leaf (Table 2). However, in all three years, the higher concentrations of SA in water deficit conditions (especially dryland) significantly increased the RWC of the *A. hirtifolium* plant (Table 3). The RWC of the leaf is known as an effective physiological marker for drought tolerance in many plants, including bread wheat [17,18]. The improvement of the leaf RWC with the application of SA, especially in conditions of low water stress in rapeseed and wheat, has been reported [19,20]. Our results show that deficit irrigation reduces RWC, but exogenous SA application improved it under stress conditions. Similar responses have been reported on *Impatiens walleriana* L. under drought stress and SA application [21]. The role of SA in maintaining the RWC rate under stress conditions also has been reported on other plants such as mustard [22] and *Brassica napus* [19]. SA increases the RWC of the plant by increasing soluble assimilates such as proline in the cell, and maintaining osmotic pressure leads to increased photosynthesis and plant growth [13,23]. However, increasing proline content in our study was not significant.

The MSI in plants under full irrigation was higher than other irrigation levels in all three years, and water scarcity significantly decreased this index. In contrast, the application of SA improved the amount of MSI in water deficit conditions (Table 2). The investigation of cell membrane degradation and its leakage ability is one of the criteria for studying the response of plants to environmental stresses such as drought [24]. It is well-known that drought stress disrupts the enzyme systems that suppress ROS, leading to increased peroxidation of membrane lipids and, consequently, damage to cell membrane stability and pigment degradation [9,12,24]. It can be stated that using this substance increases the amount of polyamines putrescine, spermidine, and spermine in the plant. These compositions can help the integrity and maintenance of the cell membrane under drought stress [25].

The accumulation of proline in plant tissues is one of the clear signs of environmental stresses, especially in plants under drought stress. This increase in proline content under stress conditions may be due to proline synthesis and the inactivation of its degradation, which protects cell membranes, proteins, and cytoplasmic enzymes, inhibits ROS, and eliminates free radicals [26]. The breakdown of proteins and carbohydrates accompanies interference in plant cell metabolism. Under these conditions, hydrolytic reactions in the plant increase, and the proteins are converted to amino acids [27]. SA causes the dynamic transport process of proline and is vital for proline’s protective role in plants. Thus, under SA treatment, glutamic-γ -semi-aldehyde (GSA) converted to pyrroline-5- carboxylate (P5C) in cytosol and chloroplasts and so increased the proline transport [28]. Mohammadi et al. found that SA application caused a significant increase in osmolytes (total carbohydrates and proline) in *Thymus kotschyanus* and *Thymus vulgaris* under drought stress conditions [10,29,30]. These results agree with our present results, especially soluble sugars (data are not shown). Likewise, increased osmolytes by SA application in barley have also been reported under combined drought and salinity stress [31] which further supports our present results. In agreement with our results, increased proline content in environmental stress conditions has been reported in *Brassica napus* [28] and *Calendula officinalis* plants when used from SA [32].

Our data indicated that the occurrence of drought stress and its increase in intensity significantly increased the activity of the SOD (Figure 2) and POD (Table 3) enzymes. This increase due to drought stress was observed in GPX activity (Table 2). The increase in these enzymes under drought stress conditions indicates their effect on reducing oxidative stress damages and their important role in the fight against free radicals [33,34]. Therefore, the following reaction of the plant is the synthesis and more significant activity of antioxidant enzymes such as the SOD enzyme to neutralize as much as possible the destructive anion of superoxide [35]. The role of SOD in a plant’s tolerance to water deficit during oxidative stress has been detected to be of great importance; this subject has been reported by many researchers [35]. Our results support those of many other authors that have reported that, with the increasing intensity of drought stress, SOD activity increases [14,36]. Another important antioxidant enzyme that plays a vital role in the breakdown and inhibition of hydrogen peroxide and its conversion to water and oxygen is the enzyme POD [29]. Increasing the activity of the POD enzyme seems to indicate the plant’s efforts to overcome hydrogen peroxide under environmental stress [37]. Increased GPX activity simultaneous with increasing the production of H_2_O_2_ in cotton and wheat exposed to drought stress indicates that this increase can lead to the uptake of ferredoxin electrons by NADP^+^, which reduces the production of superoxide [13]. This increase in activity level prevents the peroxidation of cell membrane fats under drought conditions [13].

SA increased the antioxidant activity of SOD, POD, and GPX enzymes in different irrigation levels, especially in severe drought stress (dryland and 50% of the plant’s water requirement) conditions (Figure 3 and Table 3). In agreement with the present results, in a study on maize under drought stress, SA increased antioxidant enzymes activity and corn tolerance to induce oxidative stress by active oxygen species [38]. Several studies have shown that the application of SA resulted in a positive effect by protecting plants against the oxidative damage caused by drought stress [39,40]. SOD activity was significantly increased by SA pretreatment and/or drought [28]. An increase in the activity of antioxidant enzymes might be attributable to an increase in the production of their substrates. The fact that the SA treatment of wheat plants enhances superoxide-generating enzyme activity (NADPH oxidase and extracellular POD) and antioxidant enzyme activity (SOD, CAT, and GPO) [41] supports this idea.

The activation of previously synthesized molecules and de novo synthesis is possible using SA. Dong and colleagues corroborated this by showing that SA regulates the transcription of genes for antioxidant enzymes in cucumber leaves at 8 °C. At the same time, paclobutrazol, a SA biosynthesis inhibitor, reduced the amounts of the mRNA genes SOD, CAT, and APX, altering their activity [42].

In confirmation of the results obtained in the present study, in a study on the *Impatiens walleriana* plant, it was reported that SA under drought stress conditions increased the concentration of POD in the plant [43]. In another study, POD activity was increased in wheat under drought stress. So, under drought stress and foliar application of 3 mM SA, POD increased by 14% [44]. Increased POD activity in plants under water deficit stress and SA application suggests the high demands of H_2_O_2_ quenching. SA treatment increases the activity of the antioxidant enzyme POD and reduces ROS activity [45]. SA generally improves enzymatic and photosynthetic activities [46] and balances ROS production and scavenging [47].

Decreasing the content of chlorophylls and carotenoids could be a sign of oxidative damage that targets lipids and proteins of chloroplast membrane and plant photosynthetic pigments [48]. In the current study, the plant in water shortage conditions spent most of its energy on proline producing (Figure 1) and increasing the concentration of cell sap (data are not shown). As a result, chlorophyll and carotenoid production in these conditions decreased.

SA acts as an antioxidant under drought stress. It prevents pigment damage by alleviating the destructive effects of ROS, thereby improving photosynthesis under drought stress. According to Costa and colleagues, the foliar application of SA on broccoli increased antioxidant and protectant compounds [49]. Several studies have demonstrated that exogenously applied SA maintains the integrity of chloroplast and thylakoid structures under drought stress conditions [13,50]. In reported studies, the use of SA in corn [51] and wheat [20] also increased chlorophyll content. Singh and colleagues stated that salicylic acid would increase the amount of total photosynthesis by increasing the Rubisco enzyme activity and increasing the chlorophyll content [52]. Additionally, studies have shown that salicylic acid protects the photosynthetic apparatus from oxidative stress in drought conditions [53].

A lack of available water explained the reduction in the onion yield caused by drought stress in *A. hirtifolium* plants. A lack of water in the plant reduced photosynthetic activity, inducing oxidative stress and increasing the production of ROS. Subsequently, ROS caused cell membrane degradation and instability (Table 3), and the cell membrane probably failed to perform its functions properly and suffered ion leakage. In addition, ROS degraded some photosynthetic pigments and macromolecules such as proteins and nucleic acids (Table 2; Table 3). This ROS effect caused a lack of essential proteins and enzymes needed for photosynthesis and respiration. This finally reduced photosynthesis and plant growth and yield [54]. Drought imposes photosynthetic inhibition, reduces the expansion of leaves, induces stomatal closure, and increases the levels of ROS, thereby resulting in an overall decrease in the yield of crop plants [55].

In using SA in plants, occur changes in metabolic and physiological processes. Using this material increases plant tolerance to water deficiency and decreases the adverse effects of drought stress on water status, stomatal conduction, and plant physiological activities [56]. The external application of SA in stressed plants significantly increases growth and yield. A study observed that the application of SA improved the growth and increased the yield and yield components of mung beans under drought stress [57]. In agreement with the present study, yield increases in tomatoes, cucumbers [56], and wheat [58] have also been reported under SA treatment. The improving onion yield due to the use of SA under water deficiency can be attributed to the role of this substance in stimulating plant protection mechanisms against drought stress and oxidative stress induced by it. So, the use of SA increased the content of proline (Figure 1) and other osmotic regulators (data are not shown), and it resulted in a higher activity of SOD (Figure 4) and POD enzymes (Table 3) in *A. hirtifolium*. Proline, in addition to its protective role along with other osmotic regulators, also increased the RWC of the plant (Table 3). Additionally, by degrading and removing ROS, antioxidant enzymes reduced ROS destructive effects on cell membranes, photosynthetic pigments, proteins, and other macromolecules affecting plant metabolic activities and improved the growth and yield of A. hirtifolium via maintaining membrane stability (Table 3) and photosynthetic pigments (Table 2; Table 3), improving the photosynthetic and respiratory activities of the plant. Improving plant growth due to the application of SA has also been reported in safflower [59], *Ammi visnaga* [60], and *Egletes viscosa* [46].

A study reported that the amount of allicin in garlic is varied and affected by climatic conditions and positively correlates with high temperature and drought stress [61]. In regions with high temperatures and low rainfall, *A. hirtifolium* onion has high allicin content. There were positive and negative correlations between allicin with temperature and rainfall [62]. In agreement with the present research, garlic allicin content in low rainfall environmental conditions (Egypt) has been reported more than in high rainfall areas (China) [45]. Drought stress on growth, yield, and secondary metabolites does not work the same for all plants but is contradictory. Although the defensive role of secondary metabolites is almost accepted today, the study of the mechanism of the effect of environmental stresses on the production of these materials has a complex and ambiguous picture. There is ample evidence that under stress conditions, the production of some of these compounds increases several times, but there are many reasons why this effect is not permanent. Contrary to our result in many cases, a decrease in the content of secondary metabolites has been observed under stress conditions [12,63,64].

Figure 9 showed that drought stress and increasing its intensity increased the amount of pyruvate and SA, especially in drought stress conditions (dryland and 50% of plant water requirement), decreased the amount of this trait. Drought stress on the pepper plant increased phenolic compounds and spiciness [65].

## 4. Materials and Methods

### 4.1. Experimental Arrangement

This research was carried out at a farm of Lorestan province, 5 km from Aleshtar city at a latitude of 33°86′ north, and the longitude was 48° and 26′ east. The experiment was a split-plot based on a randomized complete block design with four replications during three cropping years (2016–2017, 2017–2018, and 2018–2019). Irrigation at four levels of non-irrigation (dryland), full irrigation (100%), 75%, and 50% water requirement of the plant was the main factor, and SA at four levels of zero (control or spraying with distilled water), 0.5, 0.75, and 1 mmol was considered the sub-factor. Before planting, sampling from soil to determine physical and chemical properties of it from zero to 30 cm in depth was performed. The physical and chemical properties of the tested farm soil for two years of research are shown in Table 4. Based on the results of soil analysis, triple superphosphate fertilizers (50 kg ha^−1^), potassium sulfate (100 kg ha^−1^) before planting, and nitrogen fertilizer (urea) were used in two stages (100 kg ha^−1^) in April and May. Seedbed preparation operations included plowing in the fall, having two discs perpendicular to each other, and leveling the farm before planting. The dimensions of each main plot were 12 × 6 m, and the sub-plots were 3 × 6 m. Each sub-plot consisted of 6 rows of planting with a distance of 50 cm and a length of six meters. The distances between the plants on the planting lines were 10 cm. The distance between the main plots was two meters; the sub-plots were one meter and two meters between the blocks. *A. hirtifolium* onions were planted linearly in November. Irrigation treatments were applied from the beginning of the appearance of the flowering stem (50 days after emergence). The plant’s water requirement was determined using daily aerology data from the synoptic Aleshtar meteorological station (Table 5).

### 4.2. Irrigation and Treatment

The plant’s water requirements were calculated by measuring the evaporation and transpiration of the plant under standard conditions (ETC) according to Equation (1). ET0 indicated the evaporation and transpiration of the reference plat, and KC was the crop coefficient (Kc). The evaporation and transpiration of the reference plant were determined by applying Equation (2), while the Kc was obtained during the early, middle, and final stages of growth. The irrigation cycle was kept constant due to the calculation of the irrigation volume. The volume of water required for each plot was obtained by multiplying the daily water requirement (ETC) until irrigation, in irrigation round and area of each plot, taking into account irrigation efficiency [66]. In irrigation-deficit treatments, the actual volume of the required water was multiplied by the irrigation-deficit coefficient of each treatment. The calculated volume of water was used in each plot. Then, water was transmitted from a tank (a volume counter was installed on the exit point) into small streams placed at the sides of the plots using an irrigation hose.

ETC = ET0 × KC (1)

(2) $${\mathrm{ET}}_{0}=\frac{0.408\;\Delta \left(R_{a}-G\right)+y\frac{900}{T+273}U_{2}\left(e_{s}-e_{a}\right)}{\Delta +y\left(1+0.34U_{2}\right)}$$

In Equation (2), ET_0_ is the reference evapotranspiration (mm day^−1^); R_a_ is net radiation at the crop surface (MJ m^−2^ day^−1^); G is soil heat flux density (MJ m^−2^ day^−1^); T is mean daily air temperature at 2 m height (°C); U_2_ is wind speed at 2 m height (m s^−1^); e_s_ is saturation vapour pressure (kPa); e_a_ is actual vapor pressure (kPa); e_s_ − e_a_ is saturation vapor pressure deficit (kPa); Δ is slope vapor pressure curve (kPa °C^−1^), and y is psychrometric constant (kPa °C^−1^).

After applying the irrigation treatments, the application of SA as a leaf foliar spray with a 20 L rechargeable back sprayer Hardy model was performed. At this stage, to apply SA treatments, the desired sprayer was first calibrated and adjusted with water, and then the foliar application was performed. The plants were treated with SA after applying irrigation treatments (one week after the appearance of flower stems stage, approximately 57 days after emerging) at one time during the experiment. Weed control was performed three times by the hand-weeding method.

### 4.3. Relative Water Content (RWC)

Ritchie’s [67] method was used for measuring RWC. After applying the stress conditions, sampling was performed using scissors from the highest developed leaf, and the samples were immediately placed in ice and the laboratory. Their fresh weight was measured with a digital scale. All samples were then placed in distilled water and kept at 4 °C for 24 h. After 24 h, the saturation weight of the leaves was measured. The leaves were placed in an oven at 70 °C for another 24 h, then the dry weight of each was measured. Finally, the RWC of the leaves was obtained using the following formula.

RWC = (FW − DW)/(TW − DW) × 100 (3)

In this formula, RWC is relative water content, FW is fresh weight, TW is leaf weight in water saturation status, and DW is leaf dry weight.

### 4.4. Cell Membrane Stability Index (MSI)

To measure the cell membrane stability index, 0.1 g of the leaves was washed using distilled water and was placed in tubes containing 20 mL of distilled water. The tubes were placed in a hot water bath for 30 min at 40 °C, and their electrical conductivity was recorded by an electrical conductivity meter (EC1) after the tubes were cooled. Then, the tubes were kept in a water bath for 20 min at 100 °C, and their electrical conductivity was recorded after they were cooled (EC2). The cell membrane stability index was calculated using the following formulae in terms of percent [68].

MSI = 1 − (EC1/EC2) × 100 (4)

### 4.5. Proline Content

To measure proline from the Bates method was used [69]. In particular, 0.5 g of fresh leaves was ground and poured into a tube. In the following, 10 mL sulfo SA 3% was added, and the sample was placed in ice. The tube was centrifuged at 15,000 rpm for 10–15 min at 4 °C to separate the additional materials from the solution. Two mL of ninhydrin acid and 2 mL of pure acetic acid were added to it, and after placing in a hot water bath at 100 °C for one hour, were transferred to ice water. Then, 4 mL of toluene was added to it. After 20 s of intense shaking, the absorption of the upper dye layer was read using a spectrophotometer at a wavelength of 520 nm.

### 4.6. Antioxidant Enzyme Activity

The antioxidant activity of superoxide dismutase (SOD) was measured by the Giannopolities and Ries method [70]. In detail, 3 mL of the reaction solution was prepared for this work, including 13 mmol of methionine, 75 μmol of nitro blue tetrazolium chloride (NBT), 2 μmol of riboflavin, 50 mmol of phosphate buffer (pH = 7.8), and 50 μL of the extractable enzyme. The reaction started by turning on the fluorescent lamp. The reaction solution was placed under two 15-watt fluorescent lamps with a height of 20 cm for 1000 min at a light intensity of 1000 lux, and the reaction was terminated by turning off the lamps. The reaction solution was then covered with a black cloth until the adsorption measuring time. Adsorption was measured at 560 nm with a spectrophotometer and was recorded as unit per mg of protein.

Glutathione peroxidase (GPX) was measured by Nickel and Cunningham’s method [71]. First, 100 μL of enzyme extract was mixed with 3 mL of reading buffer containing 25 mM guaiacol and H_2_O_2_ 10 mM. A spectrophotometer read the absorbance of the sample at 470 nm in this solution. The activity of this enzyme was obtained in unit per mg of protein.

Peroxidase (POD) activity was measured by the Chance and Maehly method [72]. The reaction mixture used to measure the specific activity of this enzyme consisted of 750 μL of 70 mM hydrogen peroxide dissolved in 100 mM potassium phosphate at pH = 7, 750 μL of double-sterilized water, and 750 μL of 10 mM glycol dissolved in 2 mL of sterile water of twice-distilled. In this method, the above reaction mixture plus 20 μL of enzyme extract was poured into a 3 mL glass cuvette. POD enzyme activity was measured at 470 nm using a spectrophotometer. Its amount was recorded in μmol per minute per mg of protein.

### 4.7. Chlorophyll Content

The content of chlorophylls a, b, total, and carotenoids were measured according to the Lichtenthaler and Buschmann [73] method, and the adsorption rates for chlorophylls a, b, and carotenoids at 663, 645, and 470 nm were determined by spectrophotometer, respectively. The photosynthetic pigment concentration was calculated and expressed in mg per g of weight. In the following equations, A is the amount of light absorbed by the spectrophotometer at the specified wavelengths.

Chl_a_(ppm) = (12.25 × A663) − (2.79 × A646) (5)

Chl_b_(ppm) = (21.21 × A646) − (5.1 × A663) (6)

Chl_total_(ppm) = Chl_a_ + Chl_b_ (7)

Carotenoid = ((1000 × A470) − (1.82 × Chl_a_) − (85.02 × Chl_b_))/198 (8)

### 4.8. Yield

To measure the yield of *A. hirtifolium* (after removing two side rows and 0.5 m from the first and end of each plot), 1 square meter was harvested from the middle of the plot by determining its area by placing a wooden quadrat with an area of 1 square meter. The harvested *A. hirtifolium* was weighed digitally and recorded in g m^−2^. The harvest took place in late June.

### 4.9. Level of Allicin and Pyruvate

The allicin content of *A. hirtifolium* samples was measured by spectrophotometer. In this method, 0.5 g of *A. hirtifolium* powder was mixed with 10 mL of distilled water and, after 30 min of incubation, was centrifuged at 6000 rpm for 30 min at room temperature. Then, 1 mL of supernatant to 1 mL of buffer 4 Mercaptopyridine (50 mM Na3Po4, EDTA 2) was added. Then, the adsorption rate was determined using a spectrophotometer at a wavelength of 324 nm from the initial reaction speed of MP4. The pyruvate (degree of spiciness) of onions was assessed using the method of Schwimmer and Weston [74]. In this way, the extracts of leaves were extracted, and 30 μL of the extract was removed by the micro sampler and poured into Falcon, and 1020 μL of distilled water and 1 mL of DNPH (dinitrophenyl hydrazine) were added to it. After that, the samples were then placed in a hot water bath at 37 °C for 10 min. After this period, 5 mL of NaOH 0.5 M was added to each sample. After observing the color change, the amount of absorbance was read by a spectrophotometer with a wavelength of 420 nm, and the amount of pyruvate in μmol g^−1^ of weight fresh plant tissue was reported.

### 4.10. Statistical Analysis

After the experiments, a separate analysis of variance was performed for each year. Then, the variance uniformity test of the experimental errors obtained for three years was performed using the Hartley F test. After ensuring the uniformity of variance of the experimental errors for three years, the combined analysis of the data was performed for three years. SAS statistical software 9.1 (North Carolina State University, Raleigh, NC, USA) was used for statistical analysis and mean comparisons. The LSD method was used to compare the means of the data. Excel 2013 software (Microsoft, Albuquerque, NM, USA) was used to draw the charts.

## 5. Conclusions

The results of three-year research showed that drought stress by reducing the RWC of leaves reduced membrane stability, chlorophyll content, and finally, onion yield. However, the application of SA by increasing the proline content and improving the activity of antioxidant enzymes, or in other words, stimulating the plant protection mechanisms, improves the RWC of leaves, cell membrane stability, chlorophyll content, and plant yield under drought stress conditions and the resulting oxidative stress. This study reveals the beneficial role of SA in alleviating drought stress effects in *A. hirtifolium* and shows its importance for improving *A. hirtifolium* yield in optimal irrigation conditions. It seems that by increasing a plant’s resistance by using appropriate concentrations of SA in water deficit conditions while developing the cultivation of this plant in regions that are faced with water shortage, perhaps an acceptable yield can be produced. By cultivating this plant on the farm, the pressure on natural resources and pastures can be reduced, and their destruction can be prevented.

## Figures and Tables

**Table 1:** Combined analysis of variance of the studied physiological traits in *A. hirtifolium* under the influence of different levels of irrigation and foliar application with salicylic acid during three years of experiment (2016, 2017, and 2018).

Source	df	RWC	MSI	Proline	SOD	GPX	POD	
Year (Y)	2	98.404 **	362.73 **	6.26 **	5933.58 **	187.77 **	1680.09 **	
Block	9	6.31 ^ns^	8.59 ^ns^	0.35 ^ns^	113.53 ^ns^	37.34 ^ns^	73.81 ^ns^	
Irrigation (I)	3	2616.89 **	24,546.21 **	12.53 **	157,559.21 **	1912.15 **	92,875.41 **	
Y×I	6	4.19 ^ns^	49.32 **	0.93 ^ns^	1531.52 **	24.61 ^ns^	144.81 **	
Error I	27	3.25	7.71	0.38	152.25	31.41	75.37	
Salicylic Acid (SA)	3	132.38 **	109.51 **	1.708 ^ns^	5131.206 **	64.03 ^ns^	143.68 **	
I×SA	9	68.31 **	61.504 **	2.95 **	1586.32 **	77.47 ^ns^	316.06 **	
Y×SA	6	2.313 ^ns^	6.69 ^ns^	0.27 ^ns^	80.64 ^ns^	35.27 ^ns^	85.36 *	
Y×I×SA	18	7.03 **	14.13 **	0.24 ^ns^	179.64 ^ns^	42.32 ^ns^	100.95 **	
Error Total	108	2.101	6.68	0.2203	143.08	32.73	34.03	
CV%		2.62	3.53	4.1	3.46	6.97	2.13	
**Source**	**df**	**Chl a**	**Chl b**	**Total Chl**	**Carotenoid**	**Yield**	**Allicin**	**Pyruvate**
Year (Y)	2	0.018 **	0.01001 **	0.063 **	0.0087 **	6838.31 **	0.892 **	508.37 **
Block	9	0.0038 ^ns^	0.00035 *	0.0048 **	0.00014 ^ns^	449.386 ^ns^	0.0328 ^ns^	21.34 ^ns^
Irrigation (I)	3	0.385 **	0.073 **	0.714 **	0.047 **	10,074,753.42 **	24.45 **	2564.34 **
Y×I	6	0.00314 ^ns^	0.0024 **	0.0058 ^ns^	0.0015 **	1715.57 **	0.034 ^ns^	15.73 ^ns^
Error I	27	0.0026	0.00015	0.0031	0.000092	449.977	0.025	14.03
Salicylic Acid (SA)	3	0.0131 **	0.00195 **	0.014 **	0.0023 **	63,956.69 **	1.304 **	570.05 **
I×SA	9	0.0032 ^ns^	0.00039 **	0.0026 ^ns^	0.000807 **	14,625.449 **	0.38 **	80.9 **
Y×SA	6	0.0044 *	0.00032 **	0.0042 *	0.00083 **	1091.11 *	0.051 ^ns^	35.92 **
Y×I×SA	18	0.0013 ^ns^	0.00041 **	0.0015 ^ns^	0.00068 **	490.12 ^ns^	0.083 **	12.20 ^ns^
Error Total	108	0.00166	0.000066	0.0017	0.000081	374.85	0.036	10.55
CV%		7.04	4.29	5.37	3.17	2.05	7.42	4.85

^ns^, *, ** non-significant, significant at *p* ≤ 0.05 and *p* ≤ 0.01, respectively. RWC = relative water content, MSI = membrane stability index, SOD = superoxide dismutase, POD = peroxidase. Chl = chlorophyll.

**Table 2:** Comparison of the mean of the main effects of irrigation levels (100, 75, 50% of plant water requirement, and non-irrigation), salicylic acid foliar application (non-use, 0.5, 0.75, and 1 mmol) and year (2016, 2017, and 2018) on the studied physiological traits in *A. hirtifolium*.

	RWC (%)	MSI (%)	Proline (µmol g^−1^ FW)	Enzyme Activity			Pigments Concentration				Yield g m^−2^	Allicin (mg g^−1^ FW)	Pyruvate (µmol g^−1^ FW)
				SOD	GPX	POD	Chl a	Chl b	Chl T	Carotenoid			
				(unit mg^−1^ Protein)			(mg g^−1^ FW)						
				(µmol min^−1^)									
Year_1_	55.64 ^a^	72.85 ^b^	11.77 ^a^	346.65 ^b^	83.96 ^a^	276.49 ^a^	0.563 ^a^	0.184 ^b^	0.748 ^b^	0.289 ^a^	934.3 ^b^	2.65 ^a^	68.77 ^a^
Year_2_	56.20 ^a^	75.72 ^a^	11.35 ^b^	335.18 ^c^	81.07 ^b^	267.07 ^b^	0.596 ^a^	0.204 ^a^	0.806 ^a^	0.291 ^a^	954.9 ^a^	2.44 ^b^	63.66 ^b^
Year_3_	53.83 ^b^	71.00 ^b^	11.16 ^b^	354.32 ^a^	80.93 ^b^	275.29 ^a^	0.573 ^a^	0.181 ^b^	0.755 ^b^	0.270 ^b^	943.3 ^b^	2.63 ^a^	68.27 ^a^
I_100%_	61.96 ^a^	93.12 ^a^	11.01 ^c^	291.37 ^d^	74.36 ^d^	227.39 ^d^	0.668 ^a^	0.243 ^a^	0.912 ^a^	0.282 ^b^	1416 ^a^	1.87 ^d^	61.05 ^c^
I_75%_	60.24 ^b^	86.57 ^b^	11.02 ^c^	310.07 ^c^	79.95 ^c^	248.07 ^c^	0.608 ^b^	0.195 ^b^	0.804 ^b^	0.322 ^a^	1239 ^b^	2.12 ^c^	61.54 ^c^
I_50%_	52.81 ^c^	70.74 ^c^	11.60 ^b^	360.95 ^b^	84.51 ^b^	290.43 ^b^	0.579 ^b^	0.166 ^c^	0.744 ^c^	0.285 ^b^	677.3 ^c^	2.88 ^b^	68.35 ^b^
I_0_	45.89 ^d^	42.33 ^d^	12.07 ^a^	419.14 ^a^	89.12 ^a^	325.90 ^a^	0.455 ^c^	0.155 ^d^	0.619 ^d^	0.244 ^c^	444.9 ^d^	3.43 ^a^	76.66 ^a^
SA_1_	56.98 ^a^	75.37 ^a^	11.59 ^a^	353.45 ^a^	82.50 ^a^	276.43 ^a^	0.601 ^a^	0.194 ^a^	0.793 ^a^	0.293 ^a^	997.2 ^a^	2.38 ^c^	62.26 ^c^
SA_0.75_	55.02 ^b^	72.56 ^b^	11.15 ^b^	349.57 ^a^	82.74 ^a^	276.22 ^a^	0.575 ^ab^	0.194 ^a^	0.770 ^ab^	0.283 ^b^	937.2 ^b^	2.59 ^b^	66.74 ^b^
SA_0.5_	55.85 ^b^	72.91 ^b^	11.46 ^a^	348.28 ^a^	82.45 ^a^	274.27 ^a^	0.571 ^b^	0.192 ^a^	0.764 ^b^	0.276 ^c^	927.8 ^a^	2.53 ^b^	68.18 ^b^
SA_0_	53.05 ^c^	71.91 ^b^	11.50 ^a^	330.23 ^b^	80.27 ^a^	264.86 ^b^	0.563 ^b^	0.181 ^b^	0.752 ^b^	0.281 ^bc^	914.6 ^c^	2.78 ^a^	70.42 ^a^

Means in each column followed by similar letter(s) are not significantly different at 5% probability level as determined by the LSD test. RWC = relative water content, MSI = membrane stability index, SOD= superoxide dismutase, POD= peroxidase, Chl a, b, T = chlorophyll a, b, and total. I = irrigation, SA = salicylic acid.

**Table 3:** Comparison of the mean triple interaction of irrigation levels (100, 75, and 50% of plant water requirement and non-irrigation), salicylic acid foliar application (non-application, 0.5, 0.75, and 1 mmol) and year (2016, 2017, and 2018) on the studied physiological traits in *A. hirtifolium*.

Experiment Treatments								
Year	Irrigation Levels	Salycilic Acid Concentration	RWC	MSI	POD Activity (µmol min^−1^) (mg^−1^ Protein)	Chl b	Carotenoid	Allicin
			(%)				(mg g^−1^ FW)	
Y_1_	100%	0	64.23 ^a^	95.50 ^ab^	227.9 ^stuvwx^	0.253 ^a^	0.313 ^cdefg^	2.12 ^klmnop^
		0.5	62.42 ^abc^	92.46 ^abcde^	237.1 ^opqrstuv^	0.239 ^abc^	0.331 ^bc^	1.88 ^lnmop^
		0.75	60.35 ^abcde^	91.51 ^abcde^	232.7 ^qrstuvw^	0.252 ^a^	0.325 ^bcd^	1.95 ^lnmop^
		1	63.64 ^ab^	96.48 ^a^	226.4 ^tuvwx^	0.253 ^a^	0.327 ^bcd^	1.86 ^mnop^
	75%	0	60.14 ^abcde^	80.27 ^fgh^	238.3 ^opqrstuv^	0.181 ^de^	0.312 ^cdefgh^	2.12 ^klmnop^
		0.5	59.58 ^bcde^	87.54 ^cdef^	253.8 ^mno^	0.184 ^d^	0.278 ^jklmno^	2.20 ^ijklmno^
		0.75	60.48 ^abcde^	87.29 ^cdef^	264.3 ^lm^	0.183 ^de^	0.274 ^klmnop^	2.10 ^klmnop^
		1	61.16 ^abc^	88.28 ^bcde^	249.6 ^mnopq^	0.173 ^defgh^	0.303 ^defghij^	2.16 ^jklmnop^
	50%	0	50.50 ^hijk^	71.53 ^ijk^	278.5 ^jkv^	0.152 ^hij^	0.280 ^jklmn^	3.20 ^cdef^
		0.5	54.80 ^fgh^	66.71 ^k^	296.1 ^fghijk^	0.164 ^defghi^	0.285 ^hijklmn^	2.95 ^defgh^
		0.75	52.52 ^ghij^	70.44 ^jk^	290.0 ^ghijk^	0.163 ^defghi^	0.286 ^ghijklmn^	2.97 ^defgh^
		1	54.60 ^fghi^	72.43 ^ijk^	301.4 ^efgh^	0.158 ^efghij^	0.288 ^fghijklmn^	2.59 ^hijk^
	dryland	0	40.05 ^op^	40.90 ^no^	327.1 ^abc^	0.137 ^j^	0.234 ^qrs^	3.96 ^ab^
		0.5	47.8 ^klm^	41.46 ^mno^	330.7 ^a^	0.154 ^ghij^	0.249 ^pqrs^	3.42 ^bcd^
		0.75	48.01 ^klm^	40.49 ^no^	328.3 ^ab^	0.154 ^ghij^	0.264 ^lmnop^	3.65 ^abc^
		1	50.28 ^ijk^	42.38 ^mno^	342.1 ^a^	0.153 ^ghij^	0.279 ^jklmno^	3.21 ^cdef^
Y_2_	100%	0	63.22 ^ab^	95.79 ^ab^	219.3 ^wx^	0.259 ^a^	0.346 ^b^	1.91 ^lmnop^
		0.5	61.80 ^abc^	92.59 ^abcde^	213.3 ^x^	0.251 ^a^	0.314 ^cdef^	1.61 ^p^
		0.75	61.04 ^abc^	86.66 ^defg^	224.4 ^uvwx^	0.248 ^ab^	0.314 ^cdef^	1.64 ^op^
		1	63.22 ^ab^	97.14 ^a^	222.8 ^vwx^	0.246 ^ab^	0.377 ^a^	1.75 ^mnop^
	75%	0	60.67 ^abcd^	87.38 ^cdef^	239.1 ^opqrstuv^	0.187 ^d^	0.311 ^cdefgh^	2.06 ^klmnop^
		0.5	59.72 ^bcde^	89.45 ^abcde^	243.1 ^nopqrst^	0.236 ^abc^	0.281 ^jklmn^	1.95 ^lmnop^
		0.75	61.68 ^abc^	91.18 ^abcde^	250.9 ^mnop^	0.242 ^ab^	0.289 ^fghijklm^	2.00 ^lmnop^
		1	62.42 ^abc^	89.96 ^abcde^	249.1 ^mnopqr^	0.247 ^ab^	0.305 ^cdefghij^	2.08 ^klmnop^
	50%	0	50.56 ^hijk^	71.65 ^ijk^	278.4 ^kv^	0.168 ^defghi^	0.285 ^hijklmn^	2.96 ^defgh^
		0.5	56.55 ^defg^	74.67 ^hij^	282.8 ^ijk^	0.180 ^def^	0.287 ^fghijklmn^	2.74 ^fghi^
		0.75	53.52 ^ghij^	72.12 ^ijk^	287.2 ^hijk^	0.180 ^def^	0.286 ^hijklmn^	2.73 ^fghij^
		1	56.24 ^efg^	74.60 ^hij^	296.1 ^fghij^	0.183 ^de^	0.289 ^fghijklm^	2.44 ^hijkl^
	dryland	0	39.68 ^p^	41.29 ^mno^	305.9 ^defg^	0.136 ^j^	0.222 ^s^	3.46 ^bcd^
		0.5	49.46 ^jkl^	48.92 ^lm^	320.5 ^bcd^	0.167 ^defghi^	0.228 ^rs^	3.27 ^cdef^
		0.75	49.82 ^jkl^	47.79 ^lmn^	318.4 ^bcde^	0.173 ^defgh^	0.252 ^opqr^	3.33 ^cde^
		1	49.69 ^jkl^	50.47 ^l^	322 ^bcd^	0.167 ^defghi^	0.270 ^klmnop^	3.05 ^defg^
Y_3_	100%	0	62.73 ^abc^	93.60 ^abcd^	230.5 ^suvwx^	0.225 ^bc^	0.296 ^efghijkl^	2.26 ^ijklmn^
		0.5	60.84 ^abcd^	90.63 ^abcde^	226.8 ^stuvwx^	0.236 ^abc^	0.291 ^efghijkl^	1.91 ^lmnop^
		0.75	58.66 ^cdef^	90.29 ^abcde^	236.0 ^pqrstuvw^	0.237 ^abc^	0.317 ^cde^	1.70 ^nop^
		1	61.62 ^abc^	94.83 ^abc^	231.8 ^rstuvw^	0.216 ^c^	0.309 ^cdefghi^	1.81 ^mnop^
	75%	0	59.64 ^bcde^	78.90 ^ghi^	240.8 ^opqrstu^	0.173 ^defgh^	0.233 ^rs^	2.10 ^klmnop^
		0.5	58.34 ^cdef^	86.27 ^defg^	244.4 ^nopqrs^	0.177 ^defg^	0.263 ^mnop^	2.10 ^klmnop^
		0.75	58.64 ^cdef^	85.61 ^efg^	259.7 ^mn^	0.180 ^def^	0.266 ^lmnop^	2.25 ^ijklmn^
		1	60.47 ^abcde^	86.77 ^def^	243.8 ^nopqrst^	0.185 ^d^	0.261 ^nopq^	2.31 ^ijklm^
	50%	0	44.16 ^mno^	69.73 ^jk^	282.1 ^ijk^	0.145 ^ij^	0.279 ^jklmno^	3.20 ^cdef^
		0.5	54.58 ^fghi^	64.97 ^k^	299.6 ^fghi^	0.164 ^defghi^	0.283 ^ijklmno^	2.90 ^defgh^
		0.75	50.63 ^hijk^	68.55 ^jk^	292.1 ^ghijk^	0.167 ^defghi^	0.285 ^hijklmn^	3.10 ^cdefg^
		1	55.11 ^fg^	71.55 ^ijk^	301.0 ^efgh^	0.172 ^defgh^	0.288 ^fghijklmn^	2.77 ^efghi^
	dryland	0	41.05 ^nop^	36.51 ^o^	310.6 ^cdef^	0.152 ^hij^	0.234 ^qrs^	4.05 ^a^
		0.5	44.41 ^mno^	39.37 ^o^	343.1 ^a^	0.153 ^ghij^	0.232 ^rs^	3.47 ^bcd^
		0.75	44.88 ^mn^	38.90 ^o^	331.1 ^ab^	0.156 ^fghij^	0.235 ^qrs^	3.67 ^abc^
		1	45.60 ^lm^	39.60 ^o^	331.2 ^ab^	0.164 ^defghi^	0.248 ^pqrs^	2.60 ^ghijk^

Means in each column followed by similar letter(s) are not significantly different at 5% probability level as determined by the LSD test. RWC = relative water content, MSI = membrane stability index, POD = peroxidase, Chl b = chlorophyll b.

**Table 4:** Physical and chemical characteristics of the soil of the experiment site over three years.

Texture	Fe	Mn	Cu	Zn	K(av.)	P(av.)	pH	EC	Organic Carbon	Organic Matter	Sand	Silt	Clay	Year
	mg/kg							ds/m	%					
Silty clay loam	2.21	4.06	0.48	0.75	242.8	17.9	7.14	1.21	1.52	1.62	18	52	30	2016–2017
Silty clay loam	2.44	3.98	0.66	0.71	255.4	19.1	7.25	1.04	1.38	1.42	13	55	32	2017–2018
Silty clay loam	1.96	4.11	0.59	0.69	247.6	18.7	7.06	1.11	1.42	1.55	20	42	38	2018–2019

**Table 5:** Average monthly meteorological data of Aleshtar city meteorology station.

Month	Year	Average Temperature (°C)	Relative Humidity (%)	Maximum Wind Speed (m s^−1^)		Rainfall (mm)
		Monthly	Monthly	Monthly	Maximum	Monthly
Aban	2017	12.1	45.9	15	20	12.3
	2018	11.9	44.5	12	20	10.3
	2019	10.3	55	15	20	109.7
Azar	2017	4	57.9	11	30	53.6
	2018	4.9	61.7	17	30	39.7
	2019	6.1	72.8	14	30	123.7
Dey	2017	4.2	66.4	13	20	115.2
	2018	4.6	62.4	16	20	52.1
	2019	3.5	66.2	20	20	126.5
Bahman	2017	−0.1	71	10	21	101.1
	2018	4.5	64.8	21	21	81.4
	2019	3.7	68.9	21	21	128.3
Esfand	2017	5.4	62.2	18	30	72.9
	2018	8.2	63.8	17	30	43.2
	2019	4.1	69.1	17	30	102.4
Farvardin	2017	3.3	66.1	14	23	114.1
	2018	12	61.2	21	23	70.7
	2019	9.1	68.2	21	23	319.2
Ordibehesht	2017	16.5	55.6	11	28	35.8
	2018	13.4	71.3	23	28	208.5
	2019	13.8	59	98	28	12.4
Khordad	2017	19	46.5	10	21	0.1
	2018	19.2	55.5	18	21	6.7
	2019	20.1	51.4	17	21	0

## Data Availability

The data presented in this study are available on request from the corresponding author.

## Sample Availability

Samples of the compounds are not available from the authors.
